# Additive Effects of Maternal High Fat Diet during Lactation on Mouse Offspring

**DOI:** 10.1371/journal.pone.0092805

**Published:** 2014-03-24

**Authors:** Hisashi Masuyama, Yuji Hiramatsu

**Affiliations:** Department of Obstetrics and Gynecology, Okayama University Graduate School of Medicine, Dentistry and Pharmaceutical Sciences, Okayama, Japan; Inra, France

## Abstract

Recent reports indicated that nutrition in early infancy might influence later child health outcomes such as obesity and metabolic syndrome. Therefore, we examined the effects of maternal high fat diet (HFD) during lactation on the onset of a metabolic syndrome in their offspring. All offspring were cross-fostered by dams on the same or opposite diet to yield 4 groups: offspring from HFD-fed dams suckled by HFD-fed dams (OHH) and by control diet (CD)-fed dams (OHC) and CD-fed dams suckled by HFD-fed dams (OCH) and by CD-fed dams (OCC) mice. We examined several metabolic syndrome-related factors including body weight, blood pressure, glucose tolerance and adipocytokines. Mean body weights of OHH and OCH mice were significantly higher than those of OHC and OCC mice, respectively, with elevated systolic blood pressure. Moreover, OHH and OCH mice revealed significantly worse glucose tolerance compared with the OHC and OCC mice, respectively. Triglyceride and leptin levels were significantly increased and adiponectin levels were significantly reduced by the maternal HFD during lactation, with similar changes in leptin and adiponectin mRNA expression but without histone modifications in adipose tissues. In addition, maternal obesity induced by HFD during lactation increased and prolonged the leptin surge in the offspring and the gender differences of leptin surge were observed. Our data suggested that maternal HFD during lactation might have an additive effect on the onset of the metabolic syndrome in the offspring, irrespective of the nutritional status *in utero* through the modified leptin surge.

## Introduction

Maternal obesity in human pregnancy often results in fetal overgrowth [1], [2]. This increases the risk of the offspring developing obesity and metabolic syndrome later in life, thereby contributing to the incidence of type 2 diabetes [3]–[5]. While obesity is associated with an increased risk of almost every common complication of pregnancy, obesity in the mother may play a direct role in the transmission of an obesogenic and diabetogenic trait from generation to generation. However, several studies have shown that breast-fed infants are less likely to become obese than bottle-fed infants, suggesting that breastfeeding may attenuate the effects of the metabolic environment *in utero* in children born to obese or diabetic mothers [6]–[8].

Adipose tissue is a highly specialized endocrine and paracrine tissue that produces an array of adipocytokines such as leptin, tumor necrosis factor-α and adiponectin. It also elicits cell-mediated effects via proinflammatory and anti-inflammatory cells producing various cytokines and chemokines [9]. Such factors have local and systemic biological effects, and affect insulin sensitivity and the development of metabolic diseases [9]. Adiponectin is an adipocyte-derived hormone that acts as an antidiabetic, anti-atherogenic and anti-inflammatory adipocytokine. Decreased circulating adiponectin levels are associated with obesity, insulin resistance and type 2 diabetes [10]–[12]. Moreover, leptin plays an important role in modulating satiety and energy homeostasis [13], [14].

Obese rat and mouse dams have high levels of leptin in their circulation during pregnancy and in their milk during lactation, which may permanently affect hypothalamic functions of the offspring during development [15], [16]. At postnatal day 30 in the offspring of obese rat dams, leptin-induced suppression of food intake was found to be attenuated, demonstrating leptin resistance [15]. This suggests that the maternal increase in leptin has a negative effect on satiety in the offspring. Leptin resistance is generally associated with a state of chronic obesity in which hyperleptinemia fails to inhibit food intake and maintain lean body weight set points [17], [18]. In rodents, a leptin surge normally occurs during the second postnatal week [15], [19]. Leptin's subsequent influence on food intake depends on developmental processes that may be regulated by leptin itself at critical concentrations over a critical period.

Recent reports have indicated that nutrition in early infancy may affect later child health outcomes such as obesity and metabolic syndrome [6]–[8]. Lean offspring suckled by obese dams had increased body weight with a dysmetabolic phenotype [16]. We have previously shown that exposure to a high-fat diet (HFD) *in utero* may cause a metabolic syndrome-like phenomenon through epigenetic modifications of adipocytokine, adiponectin and leptin gene expressions [20], [21]. We therefore examined whether and how a HFD during pregnancy and lactation may affect the onset of the metabolic syndrome-like phenomenon in mouse offspring.

## Materials and Methods

### Animal Procedures

Female, 8-week-old ICR strain mice were obtained from Charles River Co. Ltd. (Tokyo, Japan). Six female pregnant mice were examined per group for all in vivo experiments. Litter sizes were same for all groups (n = 9) and we tested three male and three female offspring from each litter. Mice were kept in a temperature- and light-controlled room with free access to food and water except during tolerance tests for glucose (GTT) and insulin (ITT). After 4 weeks of feeding with a HFD (energy content 62% kcal from fat, 18% from protein and 20% from carbohydrate) or a control diet (CD; 12% kcal from fat, 28% from protein and 60% from carbohydrate; Oriental Yeast Co., Tokyo, Japan) from eight weeks old, the mice were weighed and mated. Females were checked daily for postcopulatory plugs and the presence of a plug was taken to indicate day 0.5 of pregnancy. Eight-week-old male mice for mating were fed the CD for 4 weeks before experiments or mating. Pregnant mice had free access to food and water and their food consumption was estimated by weighing the remaining food every day. Maternal weight was measured on day 20 of gestation and neonatal weight was measured on day 0.5 after birth. Offspring weights were measured every 2 weeks. All offspring were cross-fostered by dams on the same or opposite diet for 3 weeks after birth to yield 4 groups: offspring from CD-fed dams suckled by CD-fed dams (OCC mice) or by HFD-fed dams (OCH mice) and offspring from HFD-fed dams fed HFD suckled by CD-fed dams (OHC mice) or by HFD-fed dams (OHH mice). Litter sizes during suckling were culled to nine pups. All offspring were weaned onto the CD at 3 weeks of age. After weaning, the offspring had free access to food and water and their food consumption was estimated by weighing the remaining food every day. Body composition was analyzed in live mice using Echo MRI-lOO (Echo Medical Systems, Houston, TX). The systolic blood pressure of offspring was measured at 12 and 24 weeks after birth by the tail-cuff method using a Softron BP98A tail-cuff hemodynamometer (Softron, Tokyo, Japan) after the behavior and heart rate of the mice had stabilized. Blood pressure is reported as the mean of at least three measurements recorded during the same session that varied by <5%. Most of the blood pressure values were within the required range once the mice had stabilized and it was required within 10 min for each mouse. At 2, 12 or 24 weeks of age, the 6 male and 6 female offspring were anesthetized with ether prior to blood and tissue collection, and white mesenteric adipose tissue was removed, frozen immediately and stored at −70°C until analysis. Total RNA was extracted from the adipose tissue using TRIzol reagent (Life Technologies Inc., Carlsbad, CA, USA), according to the manufacturer's instructions. All animal procedures were approved by the Institutional Animal Care and Use Committee of Okayama University.

### Glucose and Insulin Tolerance Tests and Measurements of Insulin, Total Triglyceride, Adiponectin and Leptin Levels

At 12 and 24 weeks of age, male and female offspring were fasted for 16 h before receiving an intraperitoneal (i.p.) injection of d-glucose (2 g/kg body weight; Sigma-Aldrich, St. Louis, MO, USA) for the GTT (n = 6 litter per group) or for 4 h before receiving an i.p. injection of human insulin (1.0 U/kg body weight; Sigma-Aldrich) for the ITT (n = 6 litter per group). Tail blood samples were taken before and at 30, 60, 90 and 120 min after the glucose or insulin injection. Blood glucose levels were measured by the glucose oxidase method using a Medisafe automated analyzer (Termo, Tokyo, Japan). Fasting insulin, total triglyceride, adiponectin and leptin levels were determined using ELISA kits (insulin and triglycerides: Morinaga Institute of Biological Sciences Inc., Yokohama, Japan; adiponectin and leptin: R&D Systems, Inc., Minneapolis, MN, USA). Blood sample volumes for each measurement were 10–20 μl and the total sample volume collected from each mouse was less than 200 μl, which was less than 5% of total blood volume and were obtained prior to GTT. Homeostasis model assessment–insulin resistance (HOMA-IR) was calculated as the fasting insulin concentration (μU/ml) × fasting glucose concentration (mg/dl)/405 [22].

### Real-time Quantitative PCR

Real-time quantitative PCR was performed to measure mRNA levels of the leptin and adiponectin genes using a StepOne Real-time PCR System and a TaqMan RNA-to-CT Gene Kit (Applied Biosystems, Carlsbad, CA, USA). Specific primers for the mouse leptin, adiponectin and β-actin sequences were purchased from Applied Biosystems. Sequences of specific primers and accession numbers were as previously described [23]–[25]. RNA samples (25 ng) were assayed in triplicate using 15 pmol of gene-specific primers and 5 pmol of gene-specific probes. Because there were no significant differences in β-actin expression under different conditions using another housekeeping gene, GAPDH as a control (data not shown), mouse β-actin mRNA levels were measured as an internal control using a predeveloped TaqMan primer and a probe mixture (Applied Biosystems). The mRNA levels of the target genes were normalized by the β-actin mRNA levels.

### Chromatin Immunoprecipitation (ChIP) Assays

ChIP assays were performed using a ChIP assay kit (Upstate Biotechnology, Lake Placid, NY, USA) according to the manufacturer's protocol. Adipose tissues from the OCC, OCH, OHC and OHH groups (n = 6 litter, six male and six female mice for each group) at 2 and 24 weeks of age were taken for sampling. Briefly, 20-mg aliquots of frozen samples were ground in liquid nitrogen using a mortar and pestle and then washed with PBS at room temperature. The samples were then resuspended in phosphate buffered saline (PBS) and cross-linked in 1% formaldehyde for 10 min. After centrifugation, the pellet was resuspended in nucleus-swelling buffer containing protease and phosphorylation inhibitors. The nuclei were lysed in SDS lysis buffer containing protease and phosphorylation inhibitors. The chromatin was sonicated to reduce DNA fragment lengths to 0.3–1.0 kb. Chromatin was precleared in the presence of 20 μl normal serum, 2 μg salmon sperm DNA and 80 μl 25% protein A-agarose slurry. Precleared chromatin samples were subjected to immunoprecipitation at 4 °C overnight in the presence of 2 μg rabbit polyclonal antibodies against acetyl-histone H3 at lysine 9 (acetyl H3K9; Millipore, Bedford, MA, USA), dimethyl histone H3 at lysine 9 (dimethyl H3K9; Millipore) and monomethyl histone H4 at lysine 20 (monomethyl H4K20; Abcam Inc., Cambridge, MA, USA), or nonimmune rabbit IgG (Millipore). After collecting the complex by incubation with 60 μl 25% protein A-sepharose slurry and centrifugation, the beads were washed five times and the chromatin–immune complex was eluted. After reversing the crosslinking, DNA was purified and used as a template for PCR. PCR was performed using primer sets specific for the promoter region of the mouse *adiponectin* gene (positions −549 to −481) [26] and the promoter region of the mouse *leptin* gene (−181 to +20) [27].

### Statistical Analysis

Statistical analyses were performed by factorial, repeated ANOVA or Student t tests for independent groups, as appropriate, followed by Dunnett's test using StatView software, version 5.0 (Abacus Concepts, Berkeley, CA). Data are presented as mean ± SEM and P<0.05 was taken to indicate statistical significance.

## Results

### Weight, Caloric Intake, Fat Mass/Body Weight Ratio and Blood Pressure in the Male and Female Offspring

The mean maternal weight of HFD-fed pregnant mice on day 20 of gestation was significantly greater than that of CD-fed pregnant mice (49.5±2.0 vs. 37.5±1.2, p = 0.004). The weights of the neonates from HFD-fed pregnant mice on day 0.5 after birth were significantly greater than those of the neonates from CD-fed pregnant mice (1.68±0.06 vs. 1.20±0.05, p = 0.007). We observed significantly higher triglyceride (351±19 vs. 248±12, p = 0.003) and leptin (6.7±0.8 vs. 3.8±0.2, p = 0.004) levels and lower adiponectin (5.8±0.3 vs. 10.9±0.5, p = 0.002) levels in HFD-fed pregnant mice compared with the CD-fed controls on day 20 of gestation (Table 1).

**Table 1:** Maternal characteristics and neonatal weights.

Group	Maternal weight (g)	HOMA-IR	Triglyceride (mg/dl)	*Leptin (ng/dl)*	Adiponectin (μg/dl)	Neonatal weight (g)
CD	37.5±1.2	3.34±0.24	248±12	*3.8±0.2*	10.9±0.5	1.20±0.05
HFD	49.5±2.0*	4.92±0.18*	351±19*	*6.7±0.3* *	5.8±0.3*	1.68±0.06*

CD; control diet, HFD; high fat diet, mean ± SEM,

*p<0.01 vs. CD-fed pregnant mice.

**Figure 1:**
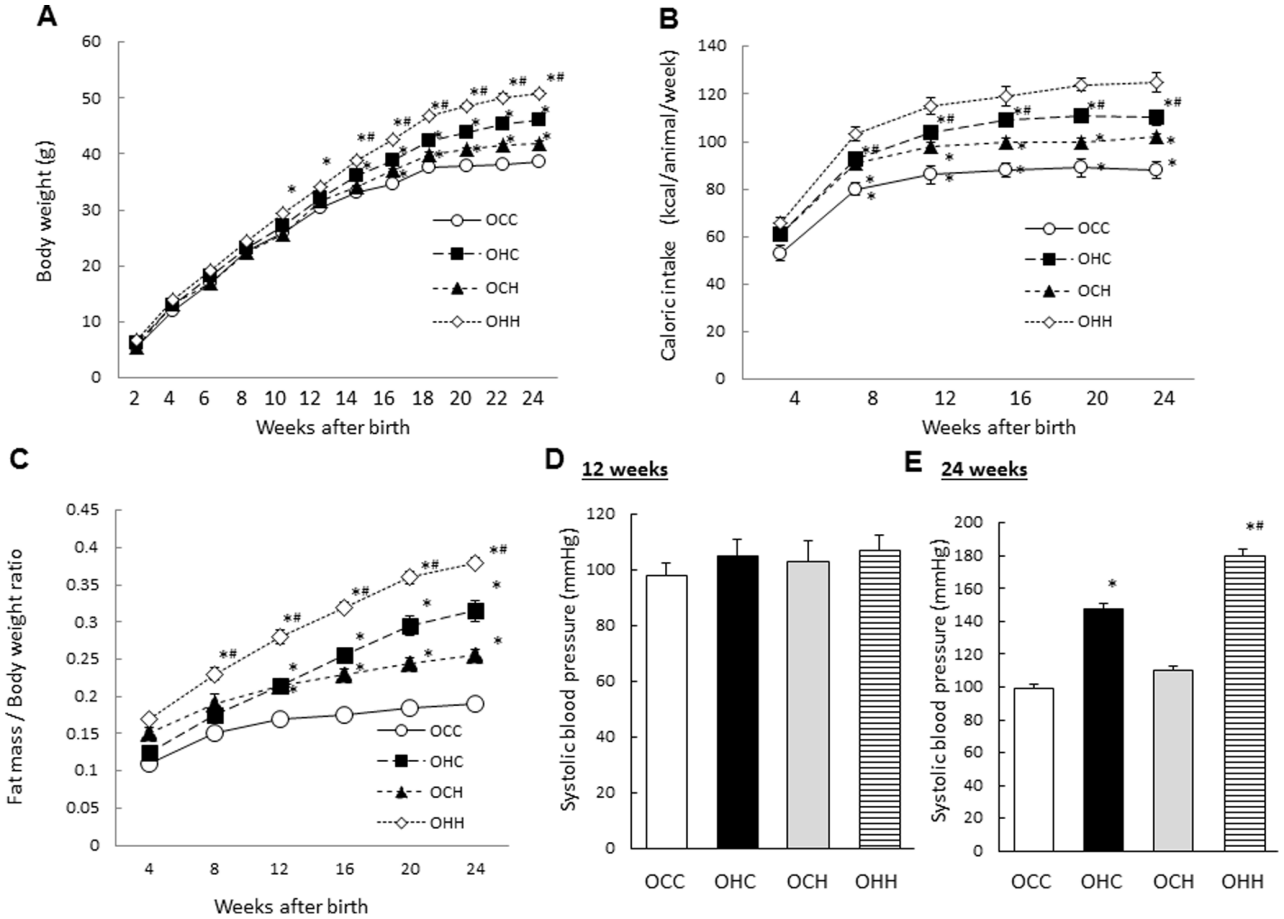
Effects of maternal HFD during lactation on the weight, caloric intake, fat mass/body weight ratio and blood pressure of the male offspring. (A) Body weight; (B) calorific intake; (C) fat mass/body ratio; and systolic blood pressure at 12 (D) and 24 (E) weeks of age. OCC: offspring from CD-fed dams suckled by CD-fed dams; OHC: offspring from HFD-fed dams suckled by CD-fed dams; OCH: offspring from CD-fed dams suckled by HFD-fed dams; OHH: offspring from HFD-fed dams suckled by HFD-fed dams. The results are shown as the mean ± SEM (*n* = 6 litter per group). *P<0.01 compared with the OCC mice and ^#^ *P*<0.01 compared with the OHC mice.

In the male offspring, the mean weights of the OHC mice were significantly higher than those of the OCC mice from 14 weeks of age. Furthermore, the mean weights of the OHH mice were higher than those of the OHC mice, and the weights of the OCH mice were higher than those of the OCC mice from 14 weeks of age (Fig. 1A). The caloric intakes of the OHC mice were significantly higher than those of the OCC mice, while the caloric intakes of the OHH mice were higher than those of the OHC mice, and the intakes of the OCH mice were higher than those of the OCC mice from 8 weeks of age (Fig. 1B). Furthermore, the gain of fat mass in the OHC mice was significantly higher than that of the OCC mice from 14 weeks of age, while fat mass gain in the OHH mice was higher than that of the OHC mice from 8 weeks of age, and fat mass gain in OCH mice was higher than that of OCC mice from 16 weeks of age (Fig. 1C). Systolic blood pressure in the OHC and OHH male mice was significantly higher than that of the OCC mice (p = 0.007, p = 0.003, respectively), while the blood pressure in the OHH mice was significantly higher than that of the OHC mice at 24 weeks of age (p = 0.008) (Fig. 1E), but not at 12 weeks of age (Fig. 1D).

**Figure 2:**
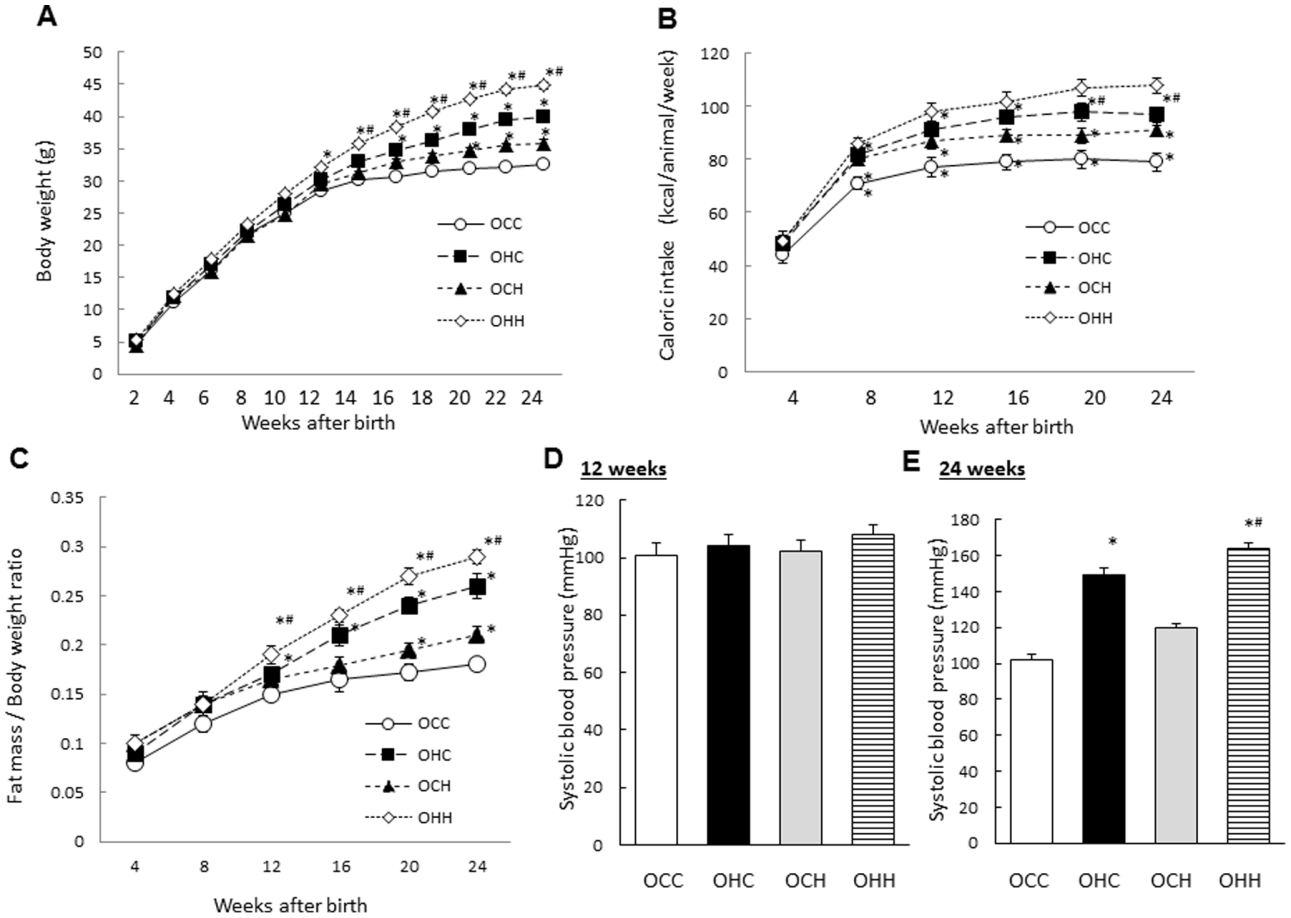
Effects of maternal HFD during lactation on the weight, caloric intake, fat mass/body weight ratio and blood pressure of the female offspring. (A) Body weight; (B) calorific intake; (C) fat mass/body ratio; and systolic blood pressure at 12 (D) and 24 (E) weeks of age. OCC: offspring from CD-fed dams suckled by CD-fed dams; OHC: offspring from HFD-fed dams suckled by CD-fed dams; OCH: offspring from CD-fed dams suckled by HFD-fed dams; OHH: offspring from HFD-fed dams suckled by HFD-fed dams. The results are shown as the mean ± SEM (*n* = 6 litter per group). *P<0.01 compared with the OCC mice and ^#^ *P*<0.01 compared with the OHC mice.

In the female offspring, there were the mild effects of maternal obesity induced by HFD on the weight, caloric intake, fat mass/body weight ratio and blood pressure compared with those in the male offspring (Fig. 2A–E).

### Effects of Maternal HFD during Lactation on Glucose Intolerance and Insulin Resistance in the Offspring

**Figure 3:**
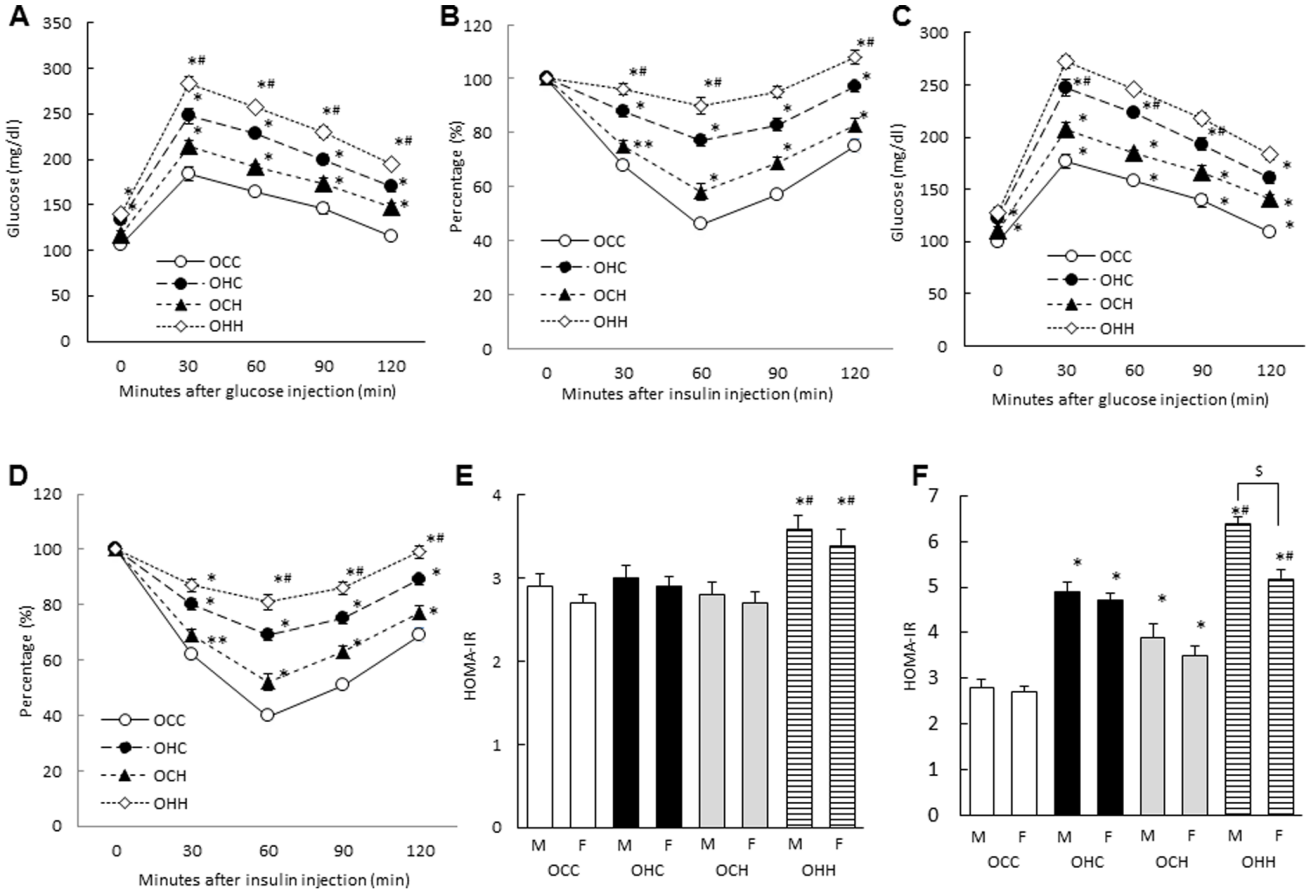
Effects of maternal HFD during lactation on glucose intolerance and insulin resistance in the offspring. GTT (A; male, C; female), ITT (B; male, D; female) at 24 weeks and HOMA-IR at 12 (E) and 24 (F) weeks of age. OCC: offspring from CD-fed dams suckled by CD-fed dams; OHC: offspring from HFD-fed dams suckled by CD-fed dams; OCH: offspring from CD-fed dams suckled by HFD-fed dams; OHH: offspring from HFD-fed dams suckled by HFD-fed dams. The results are shown as the mean ± SEM (*n* = 6 litter per group). *P<0.01, ** *P*<0.05 compared with the OCC mice and ^#^ *P*<0.01 compared with the OHC mice, ^$^ *P*<0.01 between male and female offspring in OHH group.

We performed GTTs and ITTs, measured serum insulin levels and calculated the HOMA-IR in OCC, OCH, OHC and OHH mice at 12 and 24 weeks of age to examine the effects of maternal HFD during pregnancy and lactation on glucose metabolism in the offspring. At 24 weeks of age, the OHH male mice exhibited significantly worse glucose tolerance and insulin sensitivity compared with the OCC mice. However, at this age glucose tolerance, insulin sensitivity in OHH mice were worse than those of the OHC mice, and those of the OCH mice were worse than those of the OCC mice (Fig. 3A, B). In the female offspring, there were the similar but mild effects of maternal HFD on the insulin tolerance and insulin sensitivity compared with those in the male offspring (Fig. 3C, D). The OHH male mice exhibited significantly increased HOMA-IR values compared with the OCC mice at 12 and 24 weeks of age, and there were significant differences in HOMA-IR among the other three groups at 24 weeks of age but not 12 weeks of age (Figure 3E, F). HOMA-IR was significantly increased in male offspring compared with female offspring at 24 weeks of age (p = 0.003) (Fig. 3F).

### Effects of Maternal HFD during Lactation on Serum Triglyceride and Adipocytokine Levels in the Male and Female Offspring

**Figure 4:**
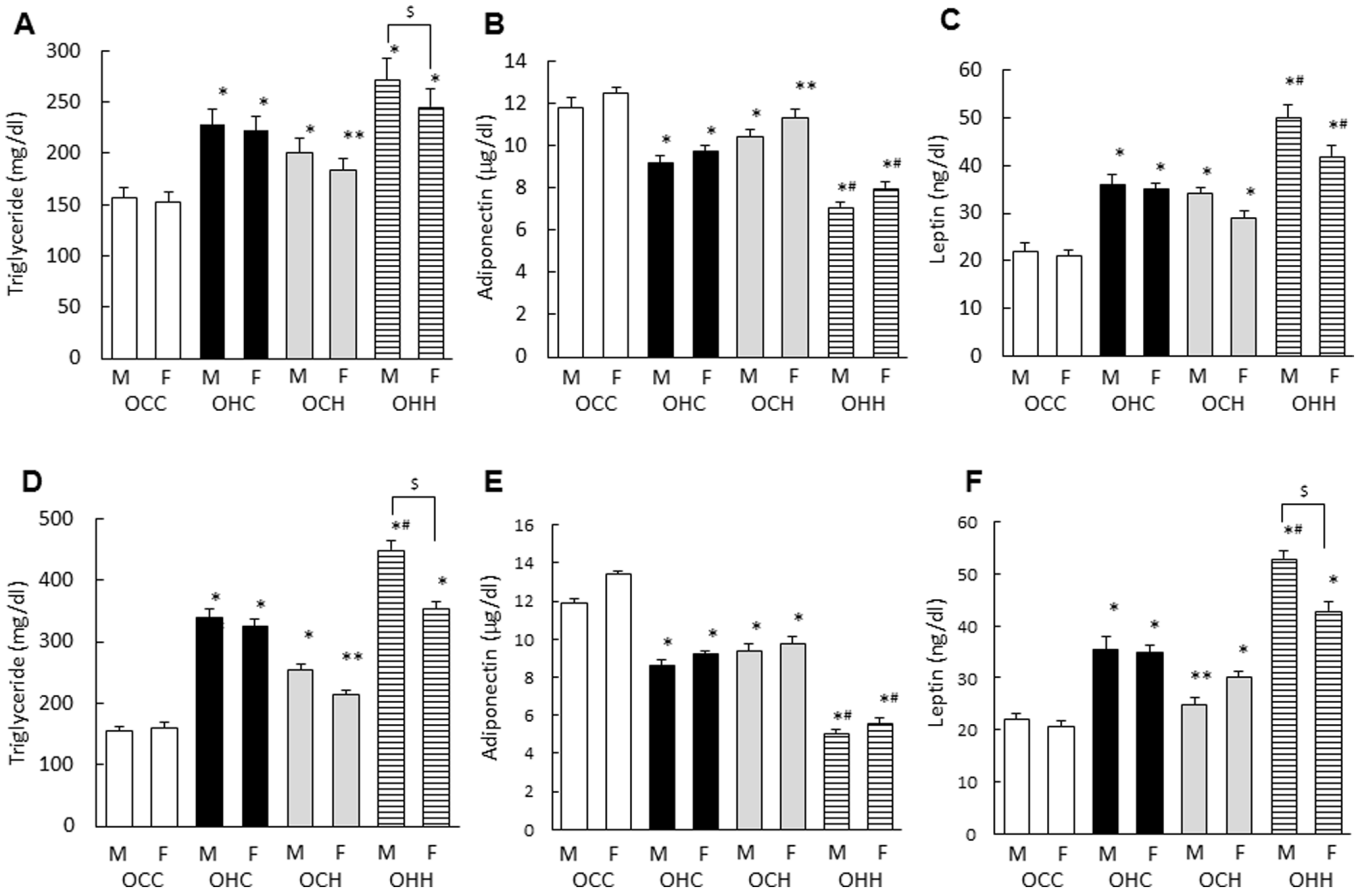
Effects of maternal HFD during lactation on serum triglyceride, adiponectin and leptin levels in the male and female offspring. Serum concentrations of total triglyceride, adiponectin and leptin at 12 (A–C) and 24 (D–F) weeks of age. OCC: offspring from CD-fed dams suckled by CD-fed dams; OHC: offspring from HFD-fed dams suckled by CD-fed dams; OCH: offspring from CD-fed dams suckled by HFD-fed dams; OHH: offspring from HFD-fed dams suckled by HFD-fed dams. The results are shown as the mean ± SEM (*n* = 6 litter per group). *P<0.01, **P<0.05 compared with the OCC mice and ^#^ *P*<0.01 compared with the OHC mice, ^$^ *P*<0.01 between male and female offspring in OHH group.

To examine the effects of maternal HFD during lactation on lipid metabolism and adipocytokine levels in the male and female offspring, serum triglyceride, leptin and adiponectin levels were examined. Total triglyceride and leptin levels were significantly higher while the adiponectin level was significantly lower in OHC mice and the OCH mice compared with the OCC mice at both 12 (Fig. 4A–C) and 24 weeks of age (Fig. 4D–F). Triglyceride and leptin were significantly higher in OHH male mice compared with OHC male mice at 24 weeks of age (Fig. 4D, F). Adiponectin at 12 and 24 weeks of age and leptin at 12 weeks of age in OHC male and female mice were significantly different compared with OHH male and female mice, respectively (Fig. 4B, C, E). In addition, there were significant gender differences of triglyceride at 12 and 24 weeks of age (p = 0.009, p = 0.003, respectively) and leptin at 24 weeks of age (p = 0.002) (Fig. 4A, D, F).

### Effects of Maternal HFD during Lactation on Leptin and Adiponectin Gene Expression Levels in the Adipose Tissue of the Male and Female Offspring

**Figure 5:**
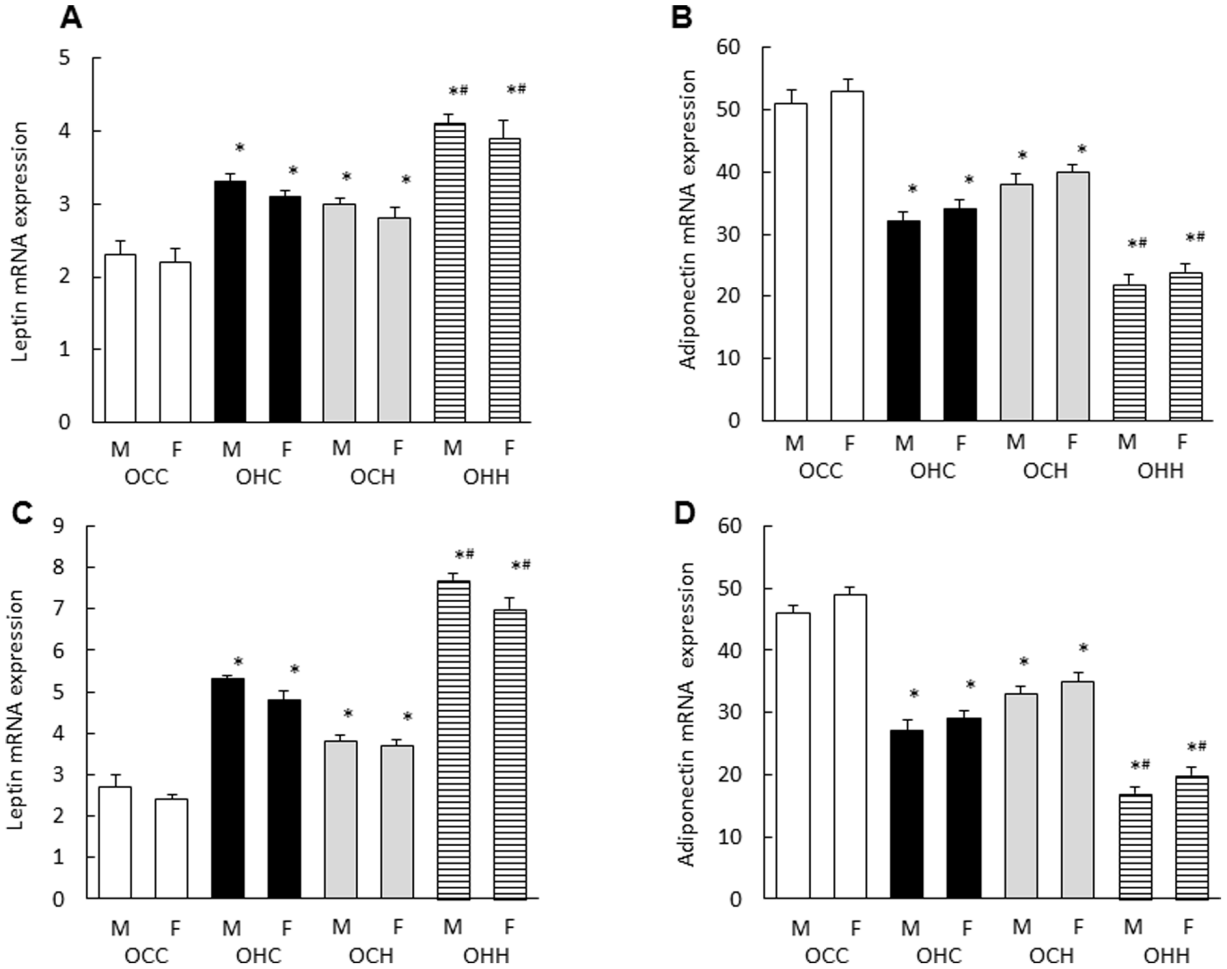
Effects of maternal HFD during lactation on leptin and adiponectin gene expression in white adipose tissue of the offspring. The white mesenteric adipose tissues were removed from male and female offspring at 12 (A, B) and 24 weeks (C, D) of age. OCC: offspring from CD-fed dams suckled by CD-fed dams; OHC: offspring from HFD-fed dams suckled by CD-fed dams; OCH: offspring from CD-fed dams suckled by HFD-fed dams; OHH: offspring from HFD-fed dams suckled by HFD-fed dams. The results are shown as the mean ± SEM (*n* = 6 litter per group). *P<0.01 compared with the OCC mice and ^#^ *P*<0.01 compared with the OHC mice.

The *leptin* gene was significantly upregulated while the *adiponectin* gene was downregulated in the white adipose tissues of OHC mice compared with the OCC mice at both 12 and 24 weeks of age (Fig. 5A–D). Maternal HFD during lactation significantly increased the *leptin* mRNA expression and decreased the *adiponectin* mRNA expression in the white adipose tissues of the OCH mice compared with those of the OCC mice, and in white adipose tissues of the OHH mice compared with those of the OHC mice(A; p = 0.004, p = 0.005, B; p = 0.002, p = 0.003, C; p = 0.002, p = 0.001, D; p = 0.003, p = 0.004, respectively) (Fig. 5A–D). There were no significant differences between male and female.

### Effects of Maternal HFD during Lactation on Modifications to H3K9 and H4K20 in the Promoter Regions of the Adiponectin and Leptin Genes in the Adipose Tissue of the Offspring

**Figure 6:**
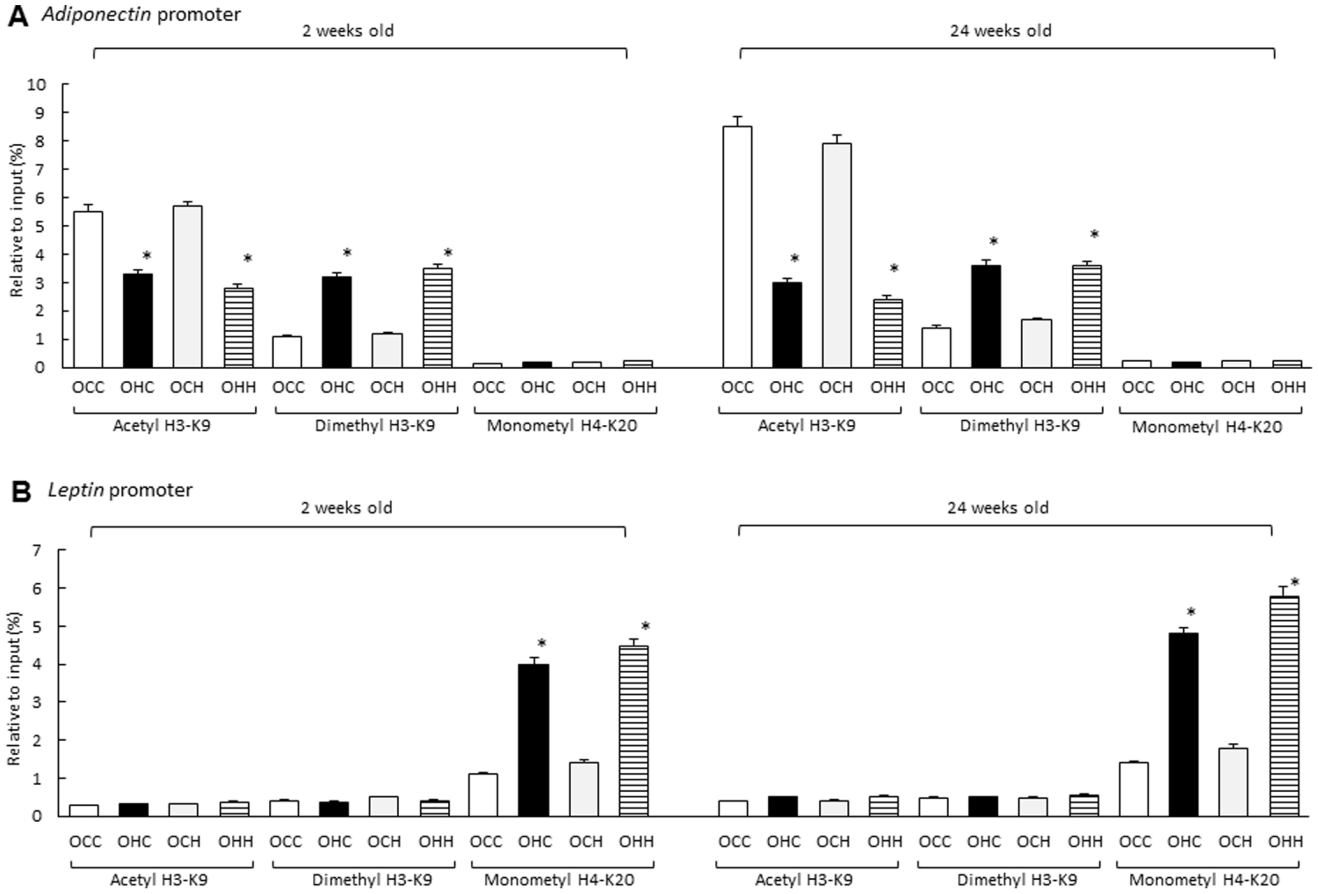
Effects of maternal HFD during lactation on modifications to H3K9 and H4K20 in the promoter regions of the *leptin* and *adiponectin* genes in the adipose tissue of the offspring. OCC: offspring from CD-fed dams suckled by CD-fed dams; OHC: offspring from HFD-fed dams suckled by CD-fed dams; OCH: offspring from CD-fed dams suckled by HFD-fed dams; OHH: offspring from HFD-fed dams suckled by HFD-fed dams. The results are shown as the mean ± SEM (*n* = 6 litter per group). *P<0.01 compared with the OCC mice.

To investigate the effects of maternal HFD during lactation on histone modifications in the promoter regions of the *adiponectin* and *leptin* genes in the adipose tissue of offspring, we performed ChIP assays using antibodies for acetyl and dimethyl H3K9 and monomethyl H4K20 at 2 and 24 weeks of age. The acetyl H3K9 level in the *adiponectin* promoter region of the OCC mice was markedly higher than that in the OHC mice, while the dimethyl H3K9 was lower in adipose tissues of OCC mice compared with those of the OHC mice. However, there were no significant differences in the modification of H3K9 between the offspring fostered by HFD-fed and CD-fed dams during lactation at either 2 or 24 weeks of age. Detection levels of monomethyl H4K20 levels were low at 2 and 24 weeks of age and there were no differences among the groups (Fig. 6A). However, the monomethyl H4K20 level was significantly increased in the *leptin* promoter region of the OHC mice compared with that of the OCC mice, while there were no significant differences in H4K20 modification between the offspring fostered by HFD-fed and CD-fed dams during lactation at either 2 or 24 weeks of age. Monomethyl H4K20 detection levels at both 2 and 24 weeks of age were weak and there were no significant differences in among the groups (Fig. 6B). There were no effects of maternal diet on the association of IgG binding with the promoter regions of *leptin* or *adiponectin* in adipose tissues and there were no significant gender differences in all promoter regions (data not shown).

### Effects of Maternal HFD during Lactation on Neonatal Leptin Concentrations and Adipose Leptin mRNA Expression in the Male and Female Offspring

**Figure 7:**
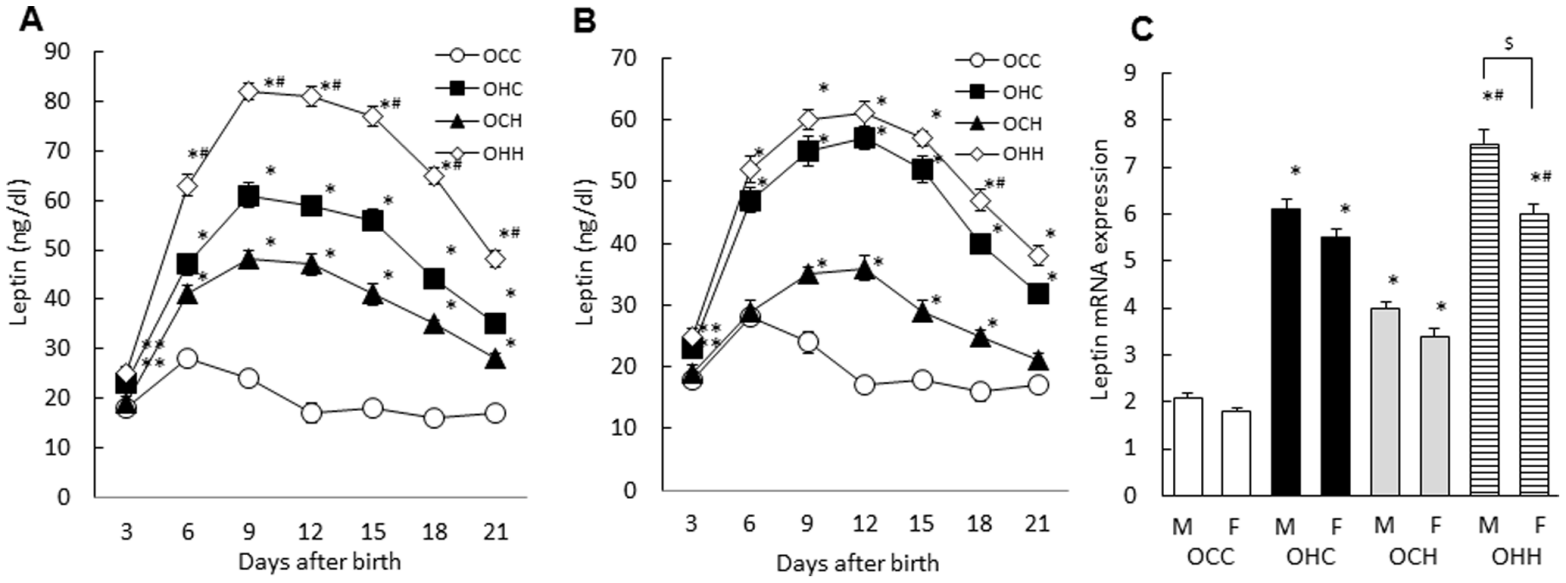
Effects of maternal HFD during lactation on neonatal leptin concentrations (A; male, B; female) and adipose leptin mRNA expression (C) in the offspring. OCC: offspring from CD-fed dams suckled by CD-fed dams; OHC: offspring from HFD-fed dams suckled by CD-fed dams; OCH: offspring from CD-fed dams suckled by HFD-fed dams; OHH: offspring from HFD-fed dams suckled by HFD-fed dams. The results are shown as the mean ± SEM (*n* = 6 litter per group). *P<0.01, **P<0.05 compared with the OCC mice and ^#^ *P*<0.01 compared with the OHC mice, ^$^ *P*<0.01 between male and female offspring in OHH group.

To examine the effects of maternal HFD during lactation on neonatal leptin concentrations and adipose leptin mRNA expression, we used the OCH and OHH mice which were suckled by HFD-fed dams and the OCC and OHC mice which were suckled by CD-fed dams for three weeks after birth. In the male offspring, the OCC mice exhibited a leptin surge during the neonatal period, while the leptin profile in the OHC mice was greatly amplified and prolonged compared with that of the OCC mice. Moreover, maternal HFD increased and prolonged the leptin surge in the OCH mice compared with that of the OCC mice and in OHH mice compared with that of the OHC mice (Fig. 7A). In contrast, there was no significant difference of leptin level between OHH mice and OHC mice in the female offspring (Fig. 7B). Leptin mRNA expression in the adipose tissue of the OHC mice on day 12 after birth was significantly higher than that of the OCC mice (p = 0.002, p = 0.003, respectively), and leptin mRNA expression in the OCH mice was significantly higher than in the OCC mice (p = 0.004, p = 0.006, respectively), and that of the OHH mice was significantly higher than that of the OHC mice (p = 0.004, p = 0.009, respectively) (Fig. 7C). In addition, the leptin mRNA expression in the OHH male mice was significantly higher compared with the OHH female mice (p = 0.005).

## Discussion

In this study, we examined whether and how maternal HFD during pregnancy and lactation affects the onset of a metabolic syndrome-like phenomenon in the male and female offspring. Body weights of mice nursed by HFD-fed dams were significantly higher than those of mice nursed by dams on the CD, while OCH weighed less than OHC, suggesting that HFD during lactation had less but additive impact on the offspring weights compared with that during gestation. This increase in body weight was accompanied by the elevated systolic blood pressure and glucose intolerance. Total triglyceride and leptin levels were significantly higher and the adiponectin level was significantly lower in mice nursed by HFD-fed dams with similar changes in leptin and adiponectin mRNA expression but without histone modifications in adipose tissues. Maternal HFD during lactation increased and prolonged the leptin surge in their offspring, irrespective of the nutritional status during gestation.

Recently, animal models of maternal overnutrition with an HFD have been developed for studies on offspring development [28]–[32]. Our previous data about the effect of HFD exposure *in utero* on the glucose and lipid metabolism of offspring were consistent with these studies [21]. These data suggest that HFD in pregnant female mice and rats causes permanent detrimental effects in body composition and metabolism in their offspring, predisposing them to the metabolic syndrome later in life even after having been weaned onto standard chow [28], [33]. We also observed dysregulation of triglyceride and adipocytokine levels ahead of worsening glucose metabolism and elevation of blood pressure [21]. Hypoadiponectinemia is associated with impaired endothelium-dependent vasodilatation in humans [34] and mice [35], as well as with insulin resistance [10]–[12]. Recent evidence indicates that leptin may represent a link between excess adiposity and increased cardiovascular sympathetic activity [36]. This suggests that aberrant production of adipocytokines in mice exposed to overnutrition *in utero* may play a role in both dysregulation of glucose and lipid metabolism and elevated blood pressure directly by metabolic imprinting or through the accumulation of fat tissue. In this study, we demonstrated that maternal HFD during lactation as well as pregnancy further aggravated the metabolic syndrome-like phenomenon in both male and female offspring by dysregulating glucose and lipid metabolism, which is consistent with the findings of a previous report [16]. We also observed that maternal HFD only during lactation (OCH mice) as well as only during pregnancy (OHC mice) affected glucose and lipid metabolism, indicating an important period of both gestation and lactation for the effect of maternal diet on metabolic change of offspring. Actually, recent report also reported that postnatal HFD during lactation in rat continued to impair glucose tolerance in the offspring in adulthood and that maternal HFD during lactation has a greater influence in determining offspring's metabolic phenotype than the HFD exposure *in utero* [37]. These data suggested that HFD and obesity during both pregnancy and lactation might play some important roles in metabolic phenotype of their offspring in adulthood.

Epigenetics can be defined as somatically heritable states of gene expression resulting from changes in chromatin structure without alterations in the DNA sequence, including DNA methylation, histone modifications and chromatin remodeling [38]. Nutrients can affect epigenetic phenomena such as DNA methylation and histone modification, thereby changing the expression of critical genes associated with physiological and pathological processes, including embryonic development [39]. In recent years, epigenetics has become an emerging issue for understanding a broad range of human diseases, such as type 2 diabetes mellitus, obesity, inflammation and neurocognitive disorders [39]. Exposure to a high fat diet *in utero* in mice induces the phenotype of type 2 diabetes and hypertension, which can be transmitted to the progeny [20], [40] and may cause a metabolic syndrome-like phenomenon through epigenetic modifications of adipocytokine, adiponectin and leptin gene expressions [21]. We have previously demonstrated that OHC mice exhibited a modification in H3K9 from methylation to acetylation in the adiponectin promoter region and methylation of H4K20 in the leptin promoter region of adipose tissue, suggesting that these histone modifications may suppress the expression of these genes in adipose tissues of OHC mice [21]. Because epigenetic changes have been shown to occur during the neonatal period [41], we examined whether maternal HFD during lactation would affect these epigenetic changes in histones in the offspring. Our data show that there were no significant differences in histone modification in the promoter regions of the *adiponectin* and *leptin* genes in adipose tissue between offspring suckled by lean and by obese dams. This suggests that the nutritional status *in utero* mainly causes the epigenetic changes in the *adiponectin* and *leptin* genes in adipose tissue. However, we need further experiments using neonate within a few days after birth to make this point clear.

Leptin levels during the perinatal period are important for the development of metabolic systems involved in energy homeostasis. In rodents, there is a postnatal leptin surge, with circulating leptin levels increasing around postnatal day (PND) 5 and peaking between PND 9 and PND 10 [15], [19]. At this time, circulating leptin acts as an important trophic factor for the development of hypothalamic circuits that control energy homeostasis and food-seeking and reward behaviors. Pharmacologically increased leptin levels in the postnatal period are associated with DNA methylation of the proopiomelanocortin promoter in the hypothalamus, which is involved in appetite and body weight control [42], and have long-term effects on metabolism [43]. These data suggest that modification of the neonatal leptin surge at specific time points may selectively affect the development of central and peripheral systems that are undergoing modifications during this period, resulting in different metabolic and behavioral outcomes. In this study, we observed that the maternal HFD during lactation increased and prolonged the leptin surge with elevated mRNA expression of leptin at adipose tissue in their offspring, regardless of the nutritional status during gestation, suggesting that enhanced leptin surge might affect the offspring metabolism. It also suggests that the adipose tissue of the offspring might be the principal source of the amplified and prolonged serum leptin surge rather than the dam's milk because previous report indicated that the ingested leptin had no effect on the leptin surge in rat neonate [15]. There was no data about the contents including leptin level in breast milk or stomach contents in our study. Several reports demonstrated that maternal high fat diet during lactation might affect the dam's milk composition including glucose, triglyceride, free fatty acid and cholesterol, and the fatty acid and glucose in milk might directly or indirectly affect hypothalamic gene expressions and development [15], [44], [45], [46], thus further analysis will be required about the effect of maternal diet during lactation on maternal endocrine function and milk composition in detail.

Because several reports demonstrated that male and female offspring responded differently to the early manipulation [47], [48], gender differences were also examined. We observed that maternal HFD during lactation but not during pregnancy had great influence on gender differences in offspring metabolism and that the peak of leptin surge during neonatal period was different between male and female in OCH and OHH groups. In our study, we observed that there were no significant differences of epigenetic changes by gender in adipocytokine genes. However, the leptin surge was significantly increased and prolonged in the OHH male offspring compared with that in the OHC male offspring, but not in the female offspring. And leptin surge in the OCH male offspring was increased and prolonged compared with the OCH female offspring. These data suggested that maternal HFD during lactation might affect the leptin surge. Postnatal leptin surge might affect long-term leptin sensitivity and control the energy homeostasis during adulthood [43], thus the gender differences of leptin surge under maternal HFD during suckling might differently affect the offspring metabolism. Further analysis will be required to show the effect and interaction among several factors including maternal diet during pregnancy, during lactation and gender in offspring metabolism. In addition, because the development of neural pathways happen post-natally in the rodent, but occur *in utero* in the human and the suckling period in a rodent, probably equates best to third trimester in human, the nutritional condition at third trimester might be more important in human.

## Conclusion

Taken together, our data suggest that maternal HFD during lactation might have an additive effect on the onset of the metabolic syndrome-like phenomenon in the offspring irrespective of the nutritional status *in utero* through the modified leptin surge. Although there is considerable evidence that breastfeeding may mitigate the adverse metabolic effects of obesity and diabetes on both mother and child [49] and had beneficial effects for the offspring in both lean and obese women, we should turn our attention to maternal obesity and overnutrition during pregnancy and lactation on their child. Because our data demonstrated here using the rodent and the diet with very high contents of fat (62% kcal from fat) might be different from the situation in human, further analysis will be required to investigate the significance of the diet during pregnancy and lactation in metabolic effects on both mother and child based on the data using the rodent.
